# The cariogenic effect of starch on oral microcosm grown within the dual constant depth film fermenter

**DOI:** 10.1371/journal.pone.0258881

**Published:** 2021-10-20

**Authors:** Jonathan M. Roberts, David J. Bradshaw, Richard J. M. Lynch, Susan M. Higham, Sabeel P. Valappil

**Affiliations:** 1 School of Dentistry, Institute of Life Course and Medical Sciences, University of Liverpool, Liverpool, United Kingdom; 2 GlaxoSmithKline Consumer Healthcare, Weybridge, United Kingdom; LSU Health Sciences Center School of Dentistry, UNITED STATES

## Abstract

Evidence on the link between starch intake and caries incidence is conflicting, therefore the cariogenicity of starch compared with sucrose was explored using a dual Constant Depth Film Fermenter (dCDFF) biotic model system. Bovine enamel discs were used as a substrate and the dCDFF was inoculated using human saliva. CDFF units were supplemented with artificial saliva growth media at a constant rate to mimic resting salivary flow rate over 14 days. The CDFF units were exposed to different conditions, 2% sucrose or 2% starch 8 times daily and either no additional fluoride or 1450 ppm F- twice daily. Bovine enamel discs were removed at intervals (days 3, 7, 10 and 14) for bacterial enumeration and enamel analysis using Quantitative Light Induced Fluorescence (QLF) and Transverse Microradiography (TMR). Results showed that in the absence of fluoride there was generally no difference in mineral loss between enamel exposed to either sucrose or starch when analysed using TMR and QLF (P > 0.05). In the presence of fluoride by day 14 there was significantly more mineral loss under starch than sucrose when analysed with TMR (P < 0.05). It was confirmed that starch and sucrose are similarly cariogenic within the dCDFF in the absence of fluoride. With the aid of salivary amylase, the bacteria utilise starch to produce an acidic environment similar to that of bacteria exposed to sucrose only. In the presence of fluoride, starch was more cariogenic which may be due to the bacteria producing a more hydrophobic intercellular matrix lowering the penetration of fluoride through the biofilm. This is significant as it indicates that the focus on sugars being the primary cause of caries may need re-evaluating and an increase in focus on carbohydrates is needed as they may be similarly cariogenic as sugars if not more so.

## Introduction

A link between starch intake and caries incidence is a topic with conflicting opinion. The Scientific Advisory Committee on Nutrition, UK reported that there is a lack of available evidence on the relationship between starch or starch rich foods and colo-rectal, oral health outcomes or cardiovascular risk factors [1]. Recent reports suggest that there is only low-quality evidence on an association between total starch intake and caries but a potential link between rapidly digestible starch and an adverse effect on oral health [2] has been noted. Other studies, including a systematic review into starch and caries, have identified a need for additional studies into the topic as until a negative or positive link has been characterised, it is not possible to state to the greater population that starch has no cariogenic affect [3] and further research is therefore essential.

To the best of our knowledge, there have been no studies into the cariogenicity of starch using the constant depth film fermenter (CDFF) model system rather than using sucrose exclusively as a means of inducing an acid response by the oral bacteria [4–6]. By employing two constant depth film fermenters in parallel as a dual setup (dCDFF) [5] it is possible to directly compare sucrose with starch whilst ensuring all other factors are equal, including artificial saliva sources and inoculum.

For this study oral bacteria were sourced from a saliva pool collected from volunteers, this was to best ensure a representative microcosm was present. The major microbes of interest for this study were *Streptococcus mutans*, *Streptococcus* spp., *Lactobacillus* spp. and *Veillonella* spp. These were chosen due to their role as primary caries pathogens [7, 8] and a link between their presence and an acidic environment [9]. The dCDFF allows control of substrate, medium composition, application of medium and depth of biofilm, which is generally set to 200 μm [10, 11] or 300 μm [12]. Bovine enamel was used throughout this study as the substrate as it is generally accepted as a suitable replacement for human enamel despite minor differences in porosity and structure [13] and has been used in other CDFF experiments [4, 12].

The loss of mineral from the bovine enamel is caused by a decrease in pH below the so called “critical pH” level [14] which results in a dissolution of minerals, including calcium and phosphate, out of the enamel and into the plaque fluid. The loss of mineral was analysed using transverse microradiography (TMR) and quantitative light induced fluorescence (QLF). TMR was chosen as it has previously been demonstrated to accurately quantify mineral loss throughout an enamel cross section showing how mineral loss changes according to depth from the surface [15]. Complementing TMR was the use of QLF, a non-destructive method to quantify mineral loss. QLF measures the loss of mineral by measuring and quantifying the changes in natural fluorescence of the enamel [16].

The aim of this study therefore was to assess whether starch is as cariogenic as sucrose within the controlled biological environment of the dCDFF, whether it affects bacterial growth and induces a structural change within the bovine enamel used as a substrate. The study will also investigate whether the efficacy of fluoride to prevent mineral loss of enamel is different between a biofilm grown under starch and one grown under sucrose.

## Materials and methods

Two separate dCDFF experiments were used in this study. The first experiment directly compared 2% sucrose with 2% starch without any additional fluoride (F-) treatment. The second experiment was setup identically to experiment 1 however 1450 ppm F- was pulsed in twice daily.

### Enamel substrate

Polished enamel discs, 5 mm in diameter, were produced from lower bovine incisors and used as the substrate for this study. Bovine mature lower incisors were extracted at an abattoir (ABP Food Group, Shrewsbury, UK) from cows under 36 months of age. They were polished using three grades of sandpaper (1200, 4000 and 7000) and painted around the sides with acid resistant nail varnish (Max Factor Crystal Clear; Proctor & Gamble, Weybridge, UK). Each disc was imaged using quantitative light induced fluorescence (QLF) to capture baseline readings. The discs were then recessed to a depth of 200 μm within Polytetrafluoroethylene (PTFE) pans, each holding five bovine enamel discs. The disc-containing pans were sterilised using 4000 Gy gamma radiation [17].

### Media preparation

An 8 L volume of artificial saliva [18, 19] was prepared for both experiments, of the composition: 1 g.L^-1^ Lab Lemco Powder (Thermo Scientific, Leicestershire, UK), 2 g.L^-1^ Yeast Extract (Sigma-Aldrich Ltd., Poole, UK), 5 g.L^-1^ Proteose Peptone (Sigma-Aldrich Ltd., Poole, UK), 2.5 g.L^-1^ Mucin from Porcine Stomach (Sigma-Aldrich Ltd., Poole, UK), 0.2 g.L^-1^ NaCl (Sigma-Aldrich Ltd., Poole, UK), 0.2 g.L^-1^ KCl (Sigma-Aldrich Ltd., Poole, UK) and 0.05 ppm fluoride, F- (as NaF, Sigma-Aldrich Ltd., Poole, UK). The 8 L volume was autoclaved at 121°C and 2200 mBar for 15 minutes. 90 u.mL^-1^ of α-amylase (from *Aspergillus oryzae*) was added aseptically after autoclaving and fully cooled. A smaller 500 mL volume of artificial saliva minus F- and α-amylase was also prepared and sterilised.

A sucrose concentration of 2% has previously been established due to its ability to induce a cariogenic response within the CDFF model [19]. As the use of starch within the CDFF is novel it was decided to use a concentration of 2% to match the sucrose source. Volumes of 2% sucrose and 2% starch were prepared and autoclaved at a lower temperature of 116°C and 1900 mBar to prevent any sugar degradation [4]. All other equipment including the CDFF units and silicon pipes were autoclaved at 121°C. For the second experiment only a solution of 1450 ppm F- (as NaF) was prepared and autoclaved at 121°C.

### dCDFF setup and inoculation

**Fig 1:**
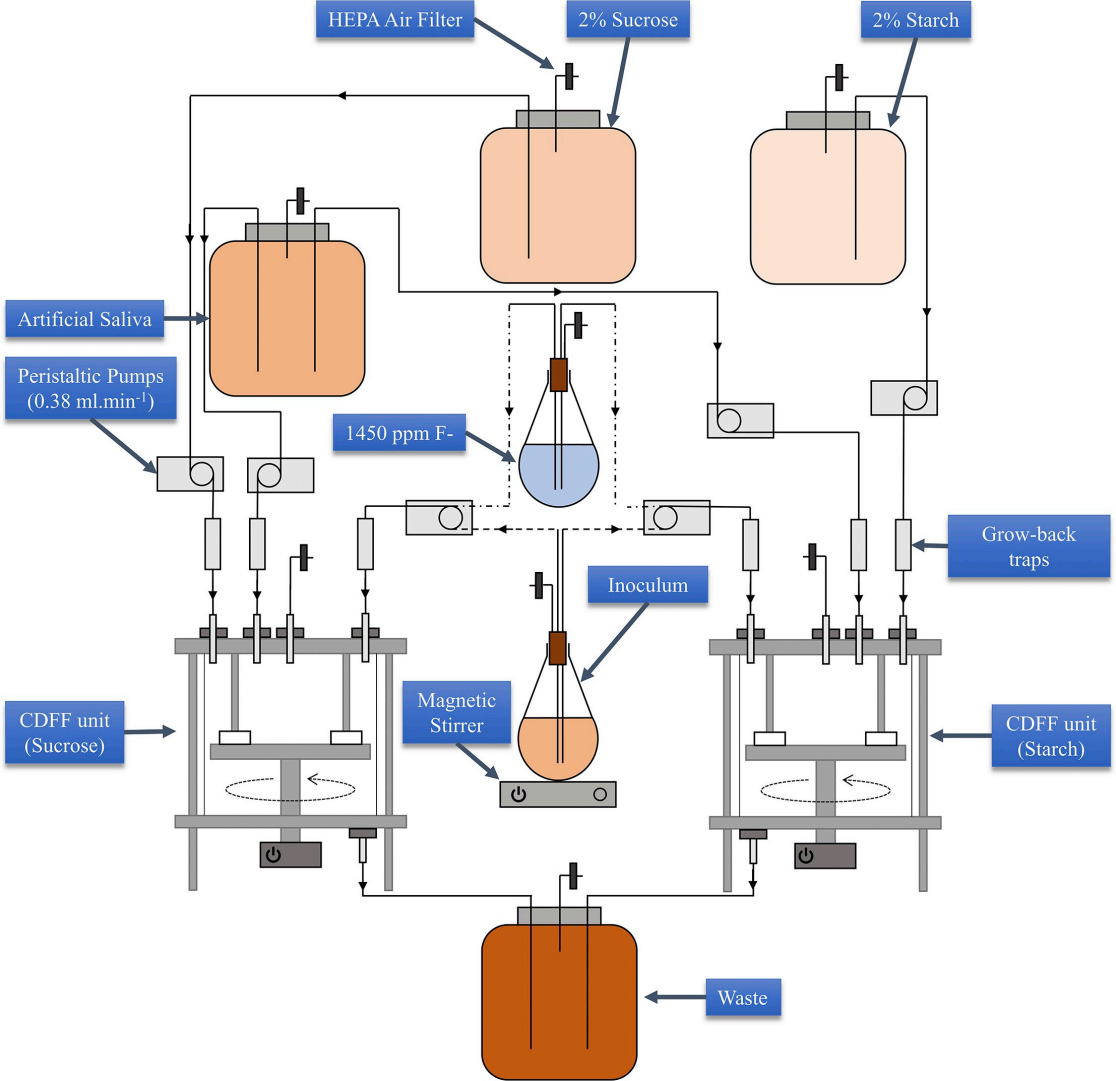
dCDFF schematic. The dCDFF was setup as shown. Each peristaltic pump was set to 0.38 ml.min^-1^ for all media into the CDFF units. All inputs incorporated a grow-back trap to prevent a cross contamination. The source of 1450 ppm F- was only used for experiment 2, all other components were the same for both experiments.

The disc-containing pans were introduced to the sterile CDFF units aseptically under laminar flow. The CDFF units and media were then setup for the experiments as shown in the Fig 1 schematic. The dCDFF was setup within an incubator set to 37°C and was an aerobic environment. Sterility of the dCDFF was maintained by filters on air exchange pipes. A pellicle was formed atop the bovine enamel discs over a 3.5-hour period at a rate of 0.38 ml.min^-1^, controlled by peristaltic pumps (101U/R MK2; Watson Marlow, Falmouth, UK), using the 8 L artificial saliva source. This was then switched off and inoculation of the dCDFF units was performed.

To provide a microcosm of oral bacteria, saliva was previously collected from 23 volunteers (ethical approval, University of Liverpool; Ref: RETH001026) who were dentate, had not taken antibiotics within 2 months and were over 18 years old [5]. Participants were informed with an information sheet outlying the purpose of the study and how their saliva was to be used. Participants who wished to continue and provide a sample gave written permission for collection of their saliva. The saliva sources were pooled, mixed 50/50 with sterile skim milk (Sigma-Aldrich Ltd., Poole, UK) to act as a cryoprotectant of the bacteria when frozen [20] and split into 1 ml aliquots for storage at -80°C. The aliquots produced may not necessarily be homogenous as observed by occasional plaque debris differences between the aliquots. However to break this down any additional processing may damage the bacteria so is generally avoided [5]. Therefore, it is not possible to directly compare colony forming units (CFUs) figures between two different experiments but is possible to discuss general trends. The two CDFF units in one experiment can be directly compared as they are inoculated with the same aliquot.

**Fig 2:**
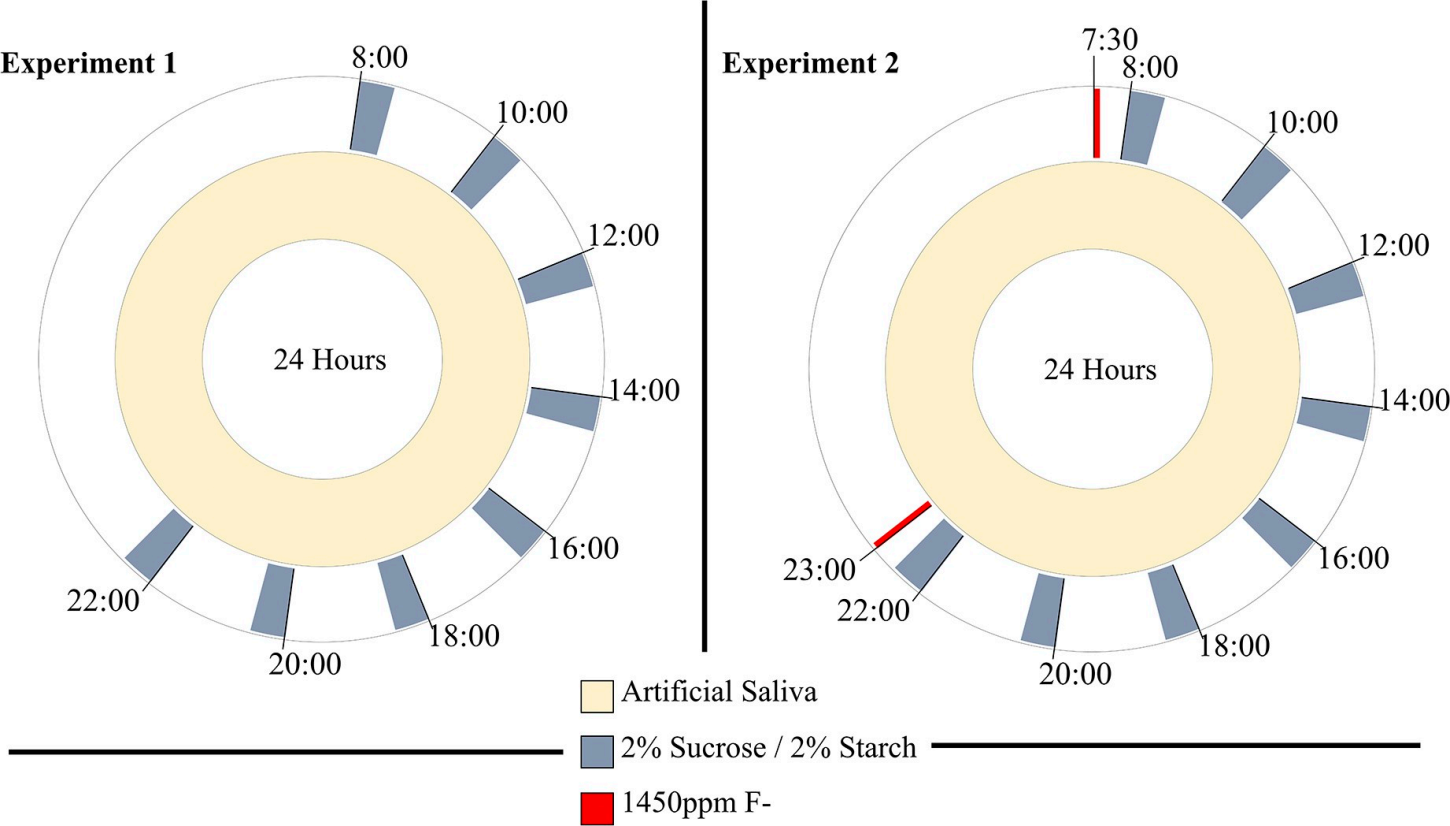
Pulsing strategy used throughout the experiments. Artificial saliva was pumped continuously whereas 2% sucrose 2% starch were pumped for 30 minutes 8 times daily. All solutions were pumped at a rate of 0.38 ml.min^-1^. Experiment 2 had additional 1450 ppm F- pulses for 2 minutes at 0.38 ml.min^-1^ before and after the daily sucrose pulses.

A thawed 1 ml aliquot of the pooled saliva was added to the 500 ml artificial saliva and pumped into the CDFF units at a rate of 0.3 ml.min^-1^ over a 16-hour period and fully depleted to inoculate both CDFF units. Once complete the 8 L artificial saliva source was restarted and the timed sources of sucrose and starch began. The carbohydrate sources were pulsed into the appropriate CDFF unit 8 times daily for 30-minute intervals at a rate of 0.38 ml.min^-1^. The 8 daily carbohydrate pulses was chosen as a previous study using 2% sucrose 8 times daily was shown to induce an acidic environment resulting in a loss of mineral from a substrate in *S*. *mutans* [21]. This was performed over 16-hours of a 24-hour cycle (Fig 2). In the second experiment F- was pulsed at 0.38 ml.min^-1^ for 2 minutes twice daily 30 minutes before the first sucrose pulse and 30 minutes after the last sucrose pulse of the day.

### Bacterial enumeration

For the enumeration of viable bacteria two PTFE pans were removed from each CDFF units on days 3, 7, 10 and 14. Two discs were removed per pan and placed in individual bijou tubes (Sterilin Ltd., Newport, UK) containing sterile 5ml PBS (Sigma-Aldrich, Poole, UK) and three sterile glass beads (3.5–4.5 mm diameter; BDH-Merk Ltd., Poole, UK) to assist the dislodging of the biofilm from the enamel surface. The bijou tubes were vortexed for 30-seconds removing the biofilm from the discs into the PBS. Serial 10-fold dilutions were performed and 10 μl of each dilution spread onto selective agars in duplicate.

*S*. *mutans* counts were enumerated using Tryptone Yeast, Cysteine agar (TYC; LabM Ltd., Bury, UK) medium with additional 3.5 mg.L^-1^ bacitracin (Sigma-Aldrich, Poole, UK). *Streptococcus* spp. enumeration was performed using Mitis Salivarius Agar (MSA; Sigma-Aldrich Ltd., Poole, UK) containing 1% potassium tellurite (Sigma-Aldrich Ltd., Poole, UK). *Lactobacillus* spp. viable counts were obtained using Rogosa agar (Sigma-Aldrich Ltd., Poole, UK). *Veillonella* spp. were enumerated using BV agar [4] which contains 15 g.L^-1^ bacto agar (Oxoid, Basingstoke, UK), 0.75 g.L^-1^ sodium thioglycollate (Sigma-Aldrich Ltd., Poole, UK), 2 mg.L^-1^ basic fuchsin (Sigma-Aldrich Ltd., Poole, UK) and 21 ml.L^-1^ of 60% Sodium Lactate (Sigma-Aldrich Ltd., Poole, UK).The pH of the medium was adjusted to 7.5 using 1 M NaOH (Sigma-Aldrich Ltd., Poole, UK) then autoclaved at 121°C. Once cooled to 47°C, 7.5 mg.L^-1^ of vancomycin (Sigma-Aldrich Ltd., Poole, UK) was added using a 0.2 μm disposable syringe filter (Sigma-Aldrich Ltd., Poole, UK).The colony forming units (CFUs) were counted to provide viable bacterial counts.

### QLF analysis

QLF measures the changes in fluorescence of the enamel. Enamel naturally fluoresces due to fluorophores within the enamel reflecting light back to the source. As mineral is lost the paths of light are disrupted resulting in less light reflected from the fluorophores [16].

Images were captured using the QLF Biluminator system (Biluminator; Inspektor Pro Research Systems, Amsterdam, Netherlands) attached to an SLR camera (Canon 660D; Canon, Tokyo, Japan) with an EF-S 60 mm *f*2.8 macro lens (Canon, Tokyo, Japan). Images were captured in a dark room with standardised settings for the blue light images: 2592x1728px, 1/40s shutter speed, *f*8.0 aperture, daylight white balance, ISO 1600. The height between sample and camera was kept consistent for all samples. Baseline images of the enamel discs were taken using the proprietary software (QLF Capture Software C3 v1.26; Inspektor Pro Research Systems, Amsterdam, Netherlands) before being placed in PTFE pans and sterilised. Once air dried after exposure to the CDFF the enamel discs were once again imaged to provide post CDFF exposure images. The images were analysed using proprietary software (QLF Analysis Software QA2 v 1.26; Inspektor Pro Research Systems, Amsterdam, Netherlands). The difference in fluorescence between the baseline image and post CDFF exposure image was used to produce a fluorescence loss value, ΔF (%), which corresponded to mineral changes in the enamel.

### TMR analysis

TMR quantifies the loss of mineral from sections of enamel by comparing the mineral content (vol%) at different depths from the surface, 0 to 100 μm in 5 μm steps, to the mineral content in the sound enamel region between the surface and dentine. This gives a ΔZ value, mineral content over depth (vol%.μm), as well as a depth of the lesion (μm) [15].

Enamel discs were mounted on ceramic discs polished side up, using Green-Stick impression compound (Kerr Corporation, California, USA) and cut into 4–5 sections of ~1.2 mm thickness using a precision diamond wire saw (Model 3241; Well Diamantrahtsagen GmbH., Mannheim, Germany). The sections were then mounted atop 11mm custom made brass anvils. Using a 50:50 slurry of nail varnish (Max factor Red Passion; Procter & Gamble, Weybridge, UK) and acetone, the sections were attached ensuring no nail varnish was between section and anvil. Once dried they were further painted around to hold the sections securely. Once set, the enamel was then polished using a diamond encrusted grinding disc (Custom-made containing 15μm sized particles; Buehler, Illinois, USA) to a 250 μm thickness. The sections were removed using acetone and remounted as previous, this time placing the recently polished side down facing the anvil. Once set, the sections were polished down to 80 μm. The sections were once again removed using acetone.

The sections were mounted onto an acetate template using double-sided sticky tape (Q-Connect, Derbystraat, Belgium) with the sound enamel sitting on the acetate and the lesion over empty space. The sections were covered with a thin X-ray film membrane to protect them. A window in the middle of the template allowed step wedge positioning for later calibration. The section-covered template was positioned atop a 12-step aluminium step wedge, with the section side touching a high-resolution x-ray film plate (HTA Enterprises Microchrome Tech Products, CA, USA) and the emulsion layer facing the x-ray source. This was exposed to a CuKα X-ray source operating at 20 mA and 20 kV for a 12-minute exposure time. The plates were then developed and fixed in solutions according to manufacturer instructions and dried before reading (Developer; EMS replacement for Kodak Developer D-19, EMS, PA, USA. Fixer; Ilford Rapid Fixer, Harman Technologies Ltd, UK)

The radiographic slides were examined using an optical microscope (BX51, Olympus, Tokyo, Japan) with a DSLR camera fitted (EOS 550D, Canon, Tokyo, Japan) through the TMR 2000 Software (version 4.0.0.23, Inspektor Research Systems BV., Amsterdam). Prior to capturing images of the sections, the exposure of the slide was calibrated using an aluminium step wedge of varying known thicknesses (25 μm steps). Following calibration, images were captured at a magnification of x20/0.4. The images were analysed using the TMR 2006 Software (version 3.0.0.17, Inspektor Research Systems BV., Amsterdam), this produced values for integrated mineral loss (ΔZ, vol%.μm) and lesion depth (μm).

### Statistical analysis

T-test statistical analyses were performed using SPSS for Windows Version 25.0 (SPSS UK Limited, Woking, UK). Calculations for mean and standard error were performed in Excel (Office 365 Version 1901: Microsoft Corporation, Redmond, WA, USA). For the statistical tests, a 95% certainty was applied, therefore the threshold for significance was P ≤ 0.05.

## Results

### Bacterial enumeration

**Fig 3:**
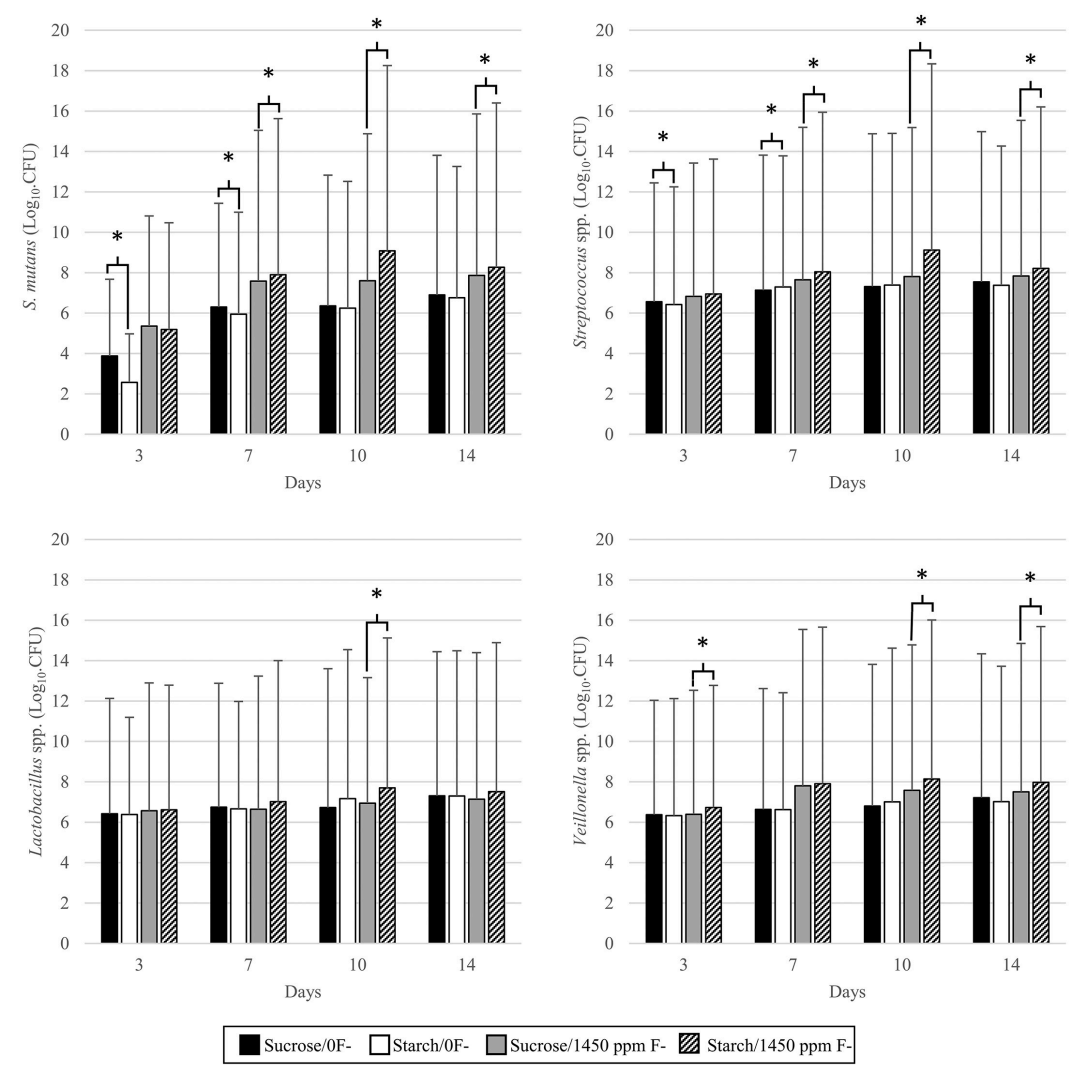
Bacterial enumeration. Viable counts of bacteria exposed to either 2% sucrose or 2% starch eight times daily. Error bars represent standard deviation of the sample set. * Denotes significance.

In the absence of additional fluoride, by day 14 for all bacterial selections (Fig 3) there was little difference between those exposed to 2% starch and those exposed to 2% sucrose. For *Lactobacillus* spp. and *Veillonella* spp. there was no statistically significant difference for all timepoints (P > 0.05). *S*. *mutans* viable counts were higher under sucrose for the duration of the experiment, however this was only significant at day 3 and (3.88 vs 2.57 log_10_.CFU.ml^-1^, P = 0.006) and day 7 (6.3 vs 5.95 log_10_.CFU.ml^-1^, P ≤ 0.001). Initially the viable counts of *Streptococcus* spp. were higher under 2% sucrose exposure at day 3 (6.56 vs 6.52 log_10_.CFU.ml^-1^, P = 0.006) but then inverted at day 7 with significantly higher counts under 2% starch (7.13 vs 7.29 log_10_.CFU.ml^-1^, P ≤0 .001). By day 10, as with *S*. *mutans*, the counts were no longer significantly different.

In the presence of twice daily pulses of 1450 ppm F-, there was generally more growth for bacteria grown under 2% starch than under 2% sucrose. Significantly more growth under starch was observed on day 3 only for *Veillonella* spp. (6.38 vs 6.73 log_10_.CFU.ml^-1^, P = 0.018). Significantly more growth of *Streptococcus* spp. under 2% starch was observed from day 7 onwards with a peak at day 10 (7.81 vs 9.12 log_10_.CFU.ml^-1^, P = 0.036) and remaining significant at day 14 (7.84 vs 8.2 log_10_.CFU.ml^-1^, P = 0.022). All 4 selections had a peak of growth under 2% starch at day 10 followed by a drop at day 14. For both experiments, all timepoints had a sample size of n = 4, 2 discs removed per pan.

### Changes in fluorescence

**Fig 4:**
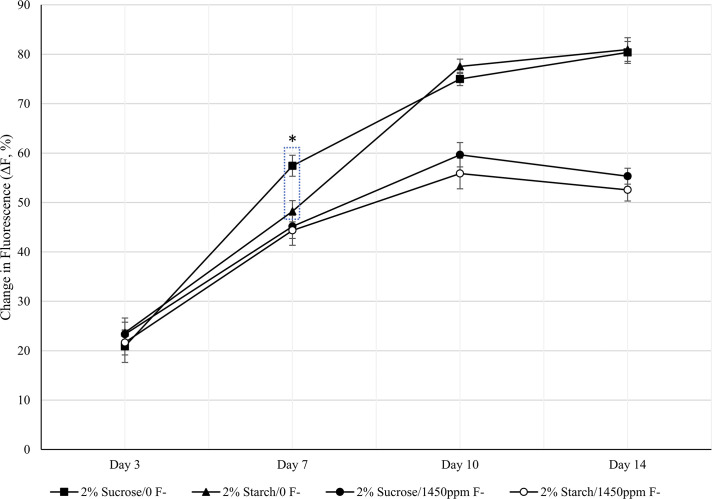
Changes in fluorescence. Change in ΔF (%) over 14-days of exposure to 2% sucrose or 2% starch eight times daily. Two CDFF units were exposed to 1450 ppm F- and two units had no additional F-. Error bars represent standard error of the sample set. * Denotes significance.

The changes in fluorescence (ΔF, %) over 14 days for enamel discs exposed to either 2% sucrose or 2% starch are shown in Fig 4. In the absence of additional fluoride, at day 3 both conditions showed a similar fluorescence loss of 20.8±3.2% for sucrose and 23.6±2.9% for starch (p = 0.534). The difference diverged at day 7 with significantly more loss under sucrose exposure (57.4±2.1 vs 48.2±2.4%, p = 0.007). The difference reduced thereafter with no significant difference observed for day 10 (p = 0.213) and day 14 (p = 0.86). For all timepoints the sample size of discs was n = 10.

When 1450 ppm fluoride was introduced twice daily there was less fluorescence loss for sucrose and starch than in the absence of fluoride. Throughout the 14 days there was no significant difference between the two conditions despite the discs exposed to sucrose having a higher fluorescence loss throughout (P > 0.05). For all time points the sample size of enamel discs was n = 10 except for a loss of a sucrose exposed disc on day 7 (n = 9).

### Mineral loss

**Fig 5:**
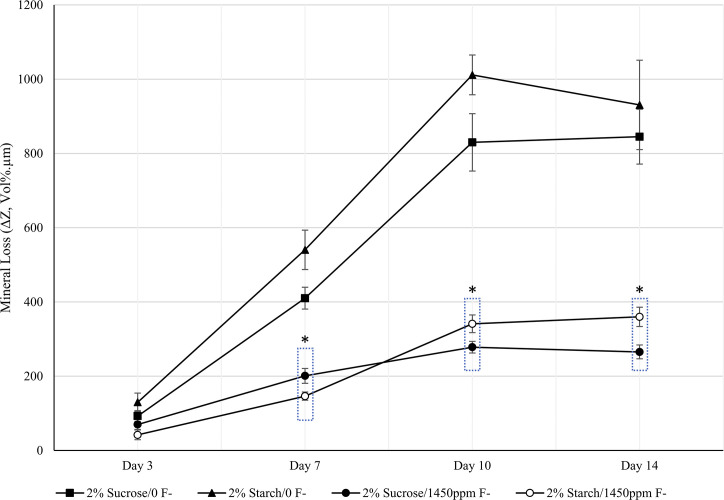
Changes in mineral loss. Mineral loss (ΔZ, Vol%.μm) over 14-days of exposure to 2% sucrose or 2% starch eight times daily. Two CDFF units were exposed to 1450 ppm F- and two units had no additional F-. Error bars represent standard error of the sample set. * Denotes significance.

The mineral loss (ΔZ, Vol%.μm) from the bovine enamel was quantified using TMR (Fig 5). Similar to the QLF analysis, in the absence of fluoride there was generally little difference between the two conditions over the 14-day duration. At day 3 there was no overall difference between discs exposed to sucrose or starch (99.6±14.7 vs 129.8±24.78 Vol%.μm, p = 0.213). The mineral loss diverged at day 7, however the mineral loss was significantly greater for discs exposed to starch (540.3±52.1 vs 410.2±29.4 Vol%.μm, p = 0.05). Beyond day 7 there was no overall difference between the two conditions at both day 10 (p = 0.069) and day 14 (p = 0.552). For all time points the sample size of enamel discs was n = 10 except for a loss of a sucrose exposed disc on day 7 (n = 9).

However, when fluoride was introduced twice daily a difference was observed from day 7 onwards. At day 7 the mineral loss data showed significantly greater mineral loss for 2% sucrose (200.95±20 vs 146.33±11 vol%.μm, p = 0.023) which then reversed for day 10 with greater mineral loss under 2% starch (278.26±15.7 vs 341.1±23.7 vol%.μm, p = 0.032) and also for day 14 (265±18.5 vs 360±25.9 vol%.μm, p = 0.001). For all time points the sample size of enamel discs was n = 10 except for a loss of a sucrose exposed disc on day 7 (n = 9).

**Fig 6:**
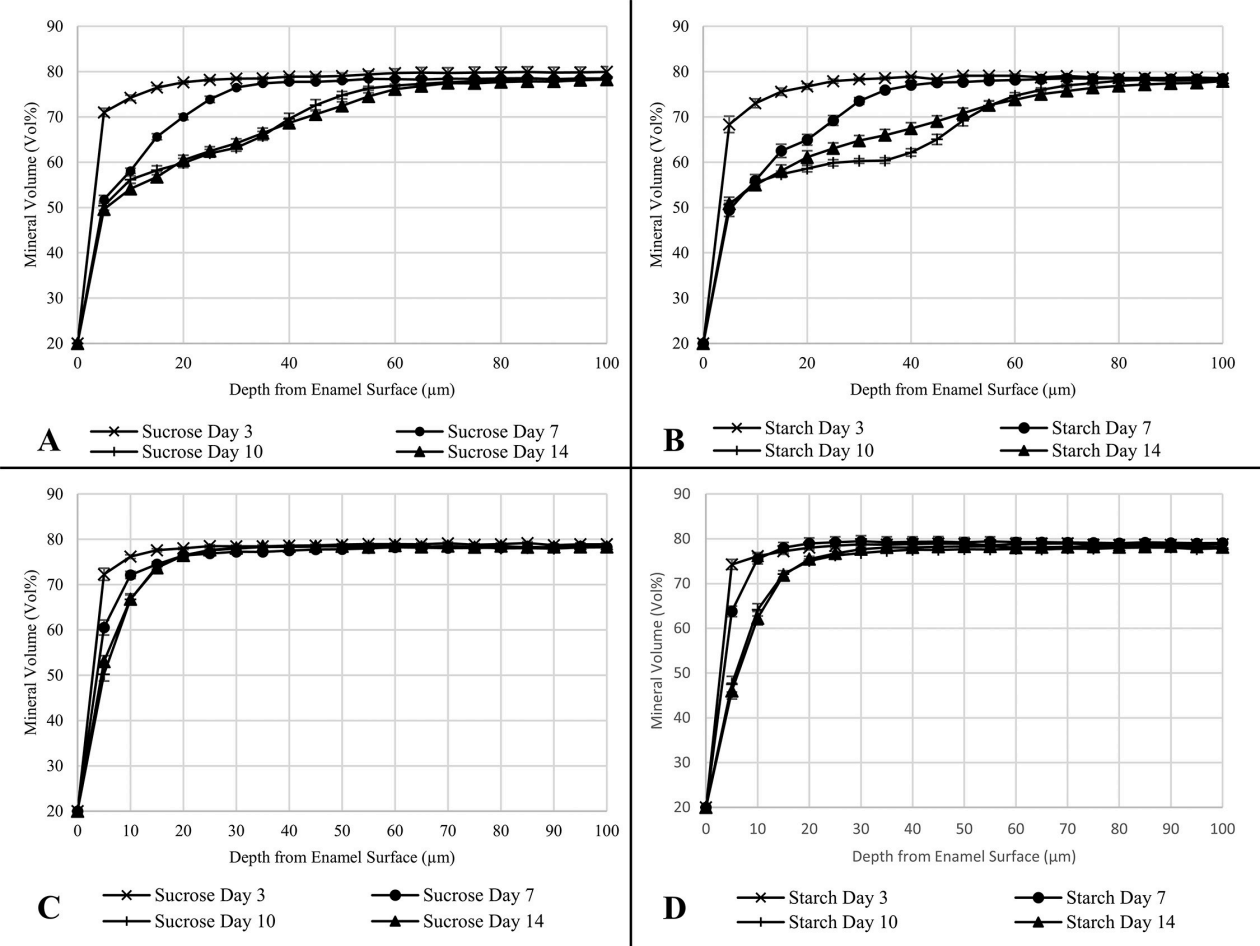
Enamel lesion profiles. Lesion profiles of bovine enamel discs exposed to either 2% sucrose or 2% starch over 14 days. (**A**) 2% Sucrose, No Fluoride. (**B**) 2% Starch, No Fluoride. (**C**) 2% Sucrose, 1450 ppm F-. (**D**) 2% Starch, 1450 ppm F-. The Lesion profiles show the change in mineral volume (Vol%) as distance from the enamel surface increases (μm).

The lesion depth profiles show the beginnings of subsurface lesions for both sucrose and starch (Fig 6A and 6B) in the absence of fluoride with similar loss under both conditions. When fluoride was introduced in the second experiment (Fig 6C and 6D) the mineral profile graphs show a similar profile for both conditions with most mineral loss at the surface and no noticeable subsurface lesions, contrasting the previous experiment where F- was absent.

## Discussion

This study using the dCDFF biological model has demonstrated that under certain conditions starch can be considered a cariogenic agent which results in a level of demineralisation comparable to sucrose. A previous study [22] investigated the growth of a microcosm supplemented by artificial saliva containing both starch and sucrose, making it difficult to ascertain whether the growth was induced by both factors or not. A study using hydroxyapatite discs in batch cultures explored the response of *S*. *mutans* to starch and sucrose during the biofilm development, showing a degree of interaction but the cariogenicity of starch alone against enamel was not investigated [23]. The dCDFF *in vitro* model employed for this study was able to investigate the cariogenicity of starch by comparing it directly with sucrose whilst closely representing of the oral environment. The previously established dCDFF model [5] was modified by using amylase at 90 u.mL^-1^ within the artificial saliva to represent the levels present within human saliva [24, 25].

### Effect of sucrose and starch on bacterial growth

This study shows that within the dCDFF model starch induces a cariogenic response by the oral bacteria that is similar to sucrose, as shown by similar levels of viable growth and mineral loss from the bovine enamel used as a substrate. The role of sucrose in oral biofilm formation is well understood and the direct relationship between sucrose intake and caries formation is well established [26]. The rapid and easy fermentation of sucrose by oral bacteria as a substrate for the synthesis of extracellular (EPS) and intracellular (IPS) polysaccharides entitles its consideration as the most cariogenic dietary carbohydrate [27]. The fermentation of sucrose leads to a pH shift of the biofilm to be more acidic resulting in caries formation [28]. Bacterial adherence to enamel is enabled by the use and production of EPS molecules that are described as having mucoid characteristics due to their sticky-like nature [29]. The EPS molecules promote structural integrity of the biofilms whilst also increasing porosity, allowing the diffusion of sucrose deeper into the biofilm which further decreases the overall pH [30]. This means the availability of glucose is integral to the establishment of the biofilm colonies and a lack thereof will reduce the overall population. The viable bacterial counts presented here in the first experiment show that there was no difference between the bacteria grown under sucrose and those under starch by day 10, implying that the bacteria were able to use starch for the formation of EPS molecules required for biofilm creation. By providing an external source of amylase in the artificial saliva, at levels comparable to human saliva (90 u.ml^-1^), glucose was made available for the formation of EPS molecules by the oral bacteria, which could not have been released in the absence of amylase [24, 25].

Twice daily pulses of 1450 ppm F- (as NaF) were introduced into the dCDFF system in the second experiment and all other conditions remained the same as the first experiment. It has been shown that F- directly inhibits enzymes within the bacteria such as enolase and F-ATPase or by increasing the permeability of the cell wall, therefore acting as an anti-microbial agent [31]. As the same concentration of F- was applied to both CDFF units equally it would be expected that the effect on bacteria growth would be equal, however this was not the case. As all bacteria isolations had higher viable counts under 2% starch, this suggests that the bacteria exposed to starch had a reduced susceptibility to F- than those grown under sucrose, therefore it may be possible that the composition of the biofilm produced from both substrates may affect the efficacy of F-.

The substrate available to the bacteria affects the composition and thickness of the biofilm, this was noted in a study which found that in the presence of starch the biofilm contained more highly branched insoluble glucans than in its absence. It also found that the combination of sucrose and starch resulted in a biofilm with greater thickness and biovolume than sucrose alone and sucrose plus glucose [32]. Distinct differences have been noted between glucans made with starch hydrolysates and those without, as well as increased adhesion by *S*. *mutans* and *Actinomyces viscosus* in the presence of starch and amylase. Therefore a change in glucans influenced by starch may affect formation of plaque and influence caries formation [33]. The presence of the additional highly branched insoluble glucans therefore may affect the overall integrity of the biofilm [34], changing the diffusion properties of the biofilm, i.e. how easily substrates and ions can move throughout the biofilm [30], resulting in an increase in protection to antimicrobial agents such as F- [8].

The differences in viable bacterial growth in experiment 2, between 2% sucrose and 2% starch exposure with additional 1450 ppm F-, may be due to highly branched glucans reducing its efficacy of F- by reducing the penetration through the biofilm. It has been shown that increased thickness of a biofilm required longer durations of F- exposure as a too short duration resulted in bacteria inhibition only at the outermost layers [35].

### Effect of sucrose and starch on mineral loss

A previous study investigating the cariogenic effect of starch vs sucrose found that in rats superinfected with *S*. *mutans* and *A*. *viscosus* starch alone was indeed cariogenic but was less so than sucrose [36]. In contrast to this, in the first experiment comparing sucrose and starch with no additional fluoride, both TMR and QLF showed similar mineral changes to the enamel under both sucrose and starch exposure. The difference between this study and the study by Firestone *et al*., may be due to the difference in microbiome composition. High levels of *S*. *mutans* has been shown to compete with other oral bacteria such as *Streptococcus sanguinis* and reduce their numbers [37]. This would reduce the amount of starch broken down as *S*. *mutans* is reliant on free α-amylase to break down starch whereas other bacteria including *S*. *sanguinis* have bound α-amylase which may aid starch breakdown [38]. Therefore by using an unaltered and representative microbiome in this study more starch was broken down for anaerobic respiration resulting in a more similar acidic environment to the sucrose exposed bacteria. In this experiment the viable counts were not significantly different for *Lactobacillus* spp. and *Veillonella* spp. exposed to either starch or sucrose. The presence of the lactic acid consumer *Veillonella* spp. and the insignificant ΔZ difference, indicate similar levels of lactic acid and both biofilms were similarly acidic.

F- is commonly used as an anti-cariogenic agent due to its ability to incorporate into the hydroxyapatite structure, increasing the acid resistance of the enamel [39], F- was used for experiment 2 at 1450 ppm to mimic the concentration in toothpastes [40]. In the presence of fluoride, the fluorescence loss of the enamel is less than in the absence of fluoride (Fig 4) for both starch and sucrose exposure. In experiment 2 when directly comparing sucrose and starch when exposed to fluoride there was no overall difference in fluorescence over the 14 days.

TMR analysis however, showed F- was acting differently against the enamel exposed to sucrose and enamel exposed to starch. This discrepancy between the TMR results and the QLF results for experiment 2 may be due to the differences in sensitivities between the two methods. It has been noted that QLF has greater sensitivity towards surface changes whereas TMR has a greater sensitivity for subsurface mineral changes [41]. As these are early subsurface lesions, TMR is therefore more suited for the measurement thereof. At day 7 there was significantly more mineral loss under sucrose, then by day 10 and into day 14 the reverse was seen with significantly greater mineral loss under starch. These results indicated that F- was less effective when in the presence of starch as the biofilm matured. The greater mineral loss for starch coincided with the greater numbers of all the bacteria selected at day 10, in particular viable *Streptococcus* spp., viable *S*. *mutans* and viable *Lactobacillus* spp., the acid producers of the biofilm. As described previously, using starch as a substrate enables the bacteria to produce more complex glucans, including soluble highly branched glucans, which may increase the structural integrity of the biofilm [34]. The increased levels of free insoluble glucans and more integral biofilm therefore may have reduced the ability of F- to diffuse through and reach the enamel [42]. If this is the case, then reduced ability to pass through the biofilm to the enamel would lower its efficacy and its ability to reduce and prevent mineral loss. The deeper lesions seen under starch exposure further indicates either a reduced ability of F- to reach the enamel as easily as when sucrose is used as a substrate or starch induces an overall more acidic environment than sucrose. This study suggests that F- at 1450 ppm may be less effective at preventing mineral loss when starch is available to the bacteria than when sucrose is available.

## Conclusion

This study has demonstrated for the first time that under dCDFF biotic model conditions, starch can be considered a cariogenic agent which results in a level of demineralisation comparable to sucrose. The dCDFF model used was able to investigate the cariogenicity of starch by comparing it directly with sucrose whilst closely representing the oral environment, therefore allowing a reliable conclusion to be drawn from the results. This conclusion will have significant implications in the field of cariology research as it indicates that the focus on sugars as the primary cause of caries may not be sufficient. Further *in vivo* research is therefore essential.

## Supporting information

S1 Dataset Underlying data used to draw conclusions in this manuscript. (XLSX)
